# Surface-Based Amplitude of Low-Frequency Fluctuation Alterations in Patients With Tinnitus Before and After Sound Therapy: A Resting-State Functional Magnetic Resonance Imaging Study

**DOI:** 10.3389/fnins.2021.709482

**Published:** 2021-11-17

**Authors:** Xuan Wei, Han Lv, Qian Chen, Zhaodi Wang, Pengfei Zhao, Chunli Liu, Shusheng Gong, Zhenghan Yang, Zhenchang Wang

**Affiliations:** ^1^Department of Radiology, Beijing Friendship Hospital, Capital Medical University, Beijing, China; ^2^Department of Otolaryngology Head and Neck Surgery, Beijing Friendship Hospital, Capital Medical University, Beijing, China

**Keywords:** tinnitus, sound therapy, surface-based analysis, ALFF, functional magnetic resonance imaging

## Abstract

This study aimed to investigate abnormal tinnitus activity by evaluating brain surface-based amplitude of low-frequency fluctuation (ALFF) changes detected by resting-state functional magnetic resonance imaging (RS-fMRI) in patients with idiopathic tinnitus before and after 24 weeks of sound therapy. We hypothesized that sound therapy could gradually return cortical local brain function to a relatively normal range. In this prospective observational study, we recruited thirty-three tinnitus patients who had undergone 24 weeks of sound therapy and 26 matched healthy controls (HCs). For the two groups of subjects, we analyzed the spontaneous neural activity of tinnitus patients by cortical ALFF and detected its correlation with clinical indicators of tinnitus. Patients’ Tinnitus Handicap Inventory (THI) scores were assessed to determine the severity of their tinnitus before and after treatment. Two-way mixed model analysis of variance and Pearson’s correlation analysis were used in the statistical analysis. Student–Newman–Keuls tests were used in the *post hoc* analysis. Interaction effects between the two groups and between the two scans revealing local neural activity as assessed by ALFF were observed in the bilateral dorsal stream visual cortex (DSVC), bilateral posterior cingulate cortex (PCC), bilateral anterior cingulate and medial prefrontal cortex (ACC and MPC), left temporo-parieto-occipital junction (TPOJ), left orbital and polar frontal cortex (OPFC), left paracentral lobular and mid cingulate cortex (PCL and MCC), right insular and frontal opercular cortex (IFOC), and left early visual cortex (EVC). Importantly, local functional activity in the left TPOJ and right PCC in the patient group was significantly lower than that in the HCs at baseline and was increased to relatively normal levels after treatment. The 24-week sound therapy tinnitus group demonstrated significantly higher ALFF in the left TPOJ and right PCC than in the tinnitus baseline group. Also, compared with the HC baseline group and the 24-week HC group, the 24-week sound therapy tinnitus group demonstrated slightly lower or higher ALFF in the left TPOJ and right PCC, and there were no differences between the 24-week sound therapy tinnitus and HC groups. Decreased THI scores and ALFF changes in the abovementioned brain regions were not correlated. Taken together, surface-based RS-fMRI can provide more subtle local functional activity to explain the mechanism of tinnitus treatment, and long-term sound therapy had a normalizing effect on tinnitus patients.

## Introduction

Tinnitus is a conscious awareness of a sound in the absence of any external acoustic stimulation, which is a major health issue in society worldwide (Bauer, 2018; Esmaili and Renton, 2018; Conlon et al., 2020). Noise trauma is the most common cause of subjective tinnitus (Grossan and Peterson, 2021), and it can trigger many related complications, such as insomnia, anxiety, depression; these complications seriously affect patients’ quality of life (Bhatt et al., 2016; Sereda et al., 2018). An increasing number of studies have shown that tinnitus, as an abnormality of the central nervous system, can lead to significant alterations in brain structure and function, such as the emotional (amygdala, anterior insula), temporofrontal/stress-regulating regions (prefrontal cortex, inferior frontal gyrus); Functional connectivity revealed increased neural coupling between several auditory areas and non-auditory areas (amygdala, cerebellum, reticular formation, hippocampus, and caudate/putamen) (Hofmeier et al., 2018; Salvi et al., 2021). These alterations are closely related to the clinical characteristics of tinnitus patients and may even be the main cause of tinnitus (Han et al., 2020; Chen et al., 2021; Wei et al., 2021). However, due to the limitations of current research methods, it is still unclear which neural pathways or brain regions play a major role in tinnitus (Vanneste et al., 2019). This may also be the reason current treatment interventions cannot achieve satisfactory results for all tinnitus patients (Langguth et al., 2013). Therefore, effective and precise treatments for certain brain regions or nerve pathways are still urgently needed.

Many methods of treating tinnitus, such as repetitive transcranial magnetic stimulation (rTMS) (Poeppl et al., 2018), drug therapy (Zenner et al., 2017), tinnitus counseling and cognitive behavioral therapy (CBT) (Langguth et al., 2013), hearing aids (Yakunina et al., 2019), cochlear implants (Olze, 2015), and tinnitus retraining therapy, have been widely used clinically (Lee et al., 2019). According to the clinical practice guidelines for tinnitus, sound therapy is one of the recommended treatment methods (Tunkel et al., 2014). During this treatment, the generated sound will be set based on tinnitus features, including its pitch, loudness, and minimum masking level. This sound reduces the contrast between the tinnitus and the environment, diminishes sensitivity to tinnitus, and promotes habituation to the tinnitus sensation (Makar et al., 2017). Narrow-band noise sound therapy has been one of the commonly used methods for the treatment of tinnitus in recent years (Henry et al., 2002). Narrow-band noise sound therapy has become the most widely used sound treatment method in our hospital and research center. In our study, narrow band sound was defined as a sound center frequency of 400 Hz or higher: the 1/3 octave band is the narrowest and the 1/2 octave band is the widest. Previous studies have demonstrated that sound therapy can alter brain function to achieve successful clinical treatment (Han et al., 2019a, b). However, previous studies were volume-based studies. The major problem in volume-based studies was that the accuracy of cortical positioning was insufficient (Coalson et al., 2018). As a result, to enhance the accuracy of cortical positioning, we adopted surface-based analysis in this study.

Different from traditional volume-based analysis, a novel 360-area surface-based cortical segmentation was applied for multimodal data using the Human Connectome Project (HCP) (Coalson et al., 2018). In this template, each cortical area has multiple characteristics, such as those representing connectivity, structure, function, auditory or visual maps (Coalson et al., 2018). Surface-based information mapping is a more sensitive measure of local information content and has better spatial selectivity and higher accuracy than other methods (Oosterhof et al., 2011). For example, surface-based analysis distinguishes between information about finger presses in the primary motor and somatosensory cortex, which can result in good spatial selectivity (Oosterhof et al., 2011). Based on the HCP template and surface-based analysis, we can extract and analyze various brain functional features, such as amplitude of low-frequency fluctuation (ALFF), regional homogeneity (ReHo), and degree centrality. In the tinnitus research field, volume-based analysis has been used in some tinnitus studies on the microstructure of the brains of tinnitus patients (Tae et al., 2018; Besteher et al., 2019). Some studies have reported microstructural changes in the brains of tinnitus patients with voxel-based morphometry (VBM) (Husain et al., 2011). Our previous study also used VBM to evaluate tinnitus patients and found that the thalamus, as a deep gray matter (GM) nucleus, was significantly different in these patients after sound therapy (Wei et al., 2020). Some studies have investigated microstructural changes such as GM and white matter (WM) volume and thickness of tinnitus patients with surface-based morphometry (SBM) (Allan et al., 2016; Meyer et al., 2016). In another study, we began to explore microstructural changes at the surface-based cortical level in tinnitus patients before and after sound therapy (Wei et al., 2021). However, no one has studied the cortical changes such as alterations in cortical ALFF, at the surface-based functional level in tinnitus patients. Pertinently, we think it is very important to fully understand the relationship between the cortical function of brain regions and tinnitus.

Amplitude of low-frequency fluctuation is a common method used to analyze resting-state functional magnetic resonance imaging (RS-fMRI) data (Kublbock et al., 2014); this form of assessment uses voxel-based analysis and focuses on the power of blood oxygen level-dependent (BOLD) signals within the low-frequency range. The advantage of this analysis lies in the ability to reflect the intensity of local neural activity and analyze the relationship between brain regions (Zang et al., 2007). ALFF has been widely used in related studies of the diagnosis and treatment of tinnitus (Chen et al., 2014, 2015; Cai et al., 2020). Previous studies have demonstrated that tinnitus is characterized by abnormal RS-fMRI findings (Han et al., 2015; Lv et al., 2016a, b, 2018b). We used graph-theoretical methods and tract-based spatial statistics (TBSS) to investigate the associations between abnormal RS-fMRI findings and clinical variables (Lv et al., 2018a; Han et al., 2019b; Chen et al., 2020). Other studies have also shown that tinnitus can cause significant changes in brain function and structure; such changes are closely related to clinical manifestations in patients (Ryu et al., 2016; Schmidt et al., 2017; Han et al., 2019a). However, these studies were limited to the volume-based level rather than the surface-based level in patients with tinnitus.

In this study, we utilized surface-based ALFF to reflect local neural activity in tinnitus patients who underwent 24 weeks of narrow-band noise sound therapy. We hypothesized that sound therapy could gradually restore local brain function to a relatively normal range, maybe involve some functional regions.

## Materials and Methods

### Standard Protocol Approval, Registration and Patient Consent

This study was approved by the ethics committees of our research institution (Beijing Friendship Hospital, Capital Medical University, 2016-P2-012). Written informed consent was obtained from all study subjects.

### Subjects

All patients and healthy volunteers were recruited from our institution. In this study, thirty-three patients with idiopathic tinnitus were enrolled. There were two patients with right laterality, five patients with left laterality, the rest had bilateral laterality. The tinnitus sound was described by patients as a persistent, low- or high-pitched sound in one or both ears. Sound more than 4 kHz is considered high-frequency in clinical research. The inclusion criteria were as follows: 18–65 years old, persistent idiopathic tinnitus (persistent for ≥6 and ≤48 months), right handedness, no significant hearing loss, and no history of associated brain diseases confirmed by conventional MRI. The exclusion criteria included neurological signs and/or a history of neurological disease; cardiovascular disease; pulsatile tinnitus, Meniere’s disease, sudden deafness, or otosclerosis; claustrophobia; and inability to pitch-match their tinnitus. Twenty-six age-, sex-, education-, and handedness-matched healthy control (HC) subjects were enrolled as controls. The characteristics of the subjects are presented in Table 1.

**TABLE 1:** Demographic and clinical characteristics of participants.

**Characteristics**	**Tinnitus patients (baseline, *n* = 33)**	**Tinnitus patients (24th week, *n* = 33)**	**Healthy controls (baseline, *n* = 26)**	**Healthy controls (24th week, *n* = 26)**	***P* value**
Age (years,$\bar{x}$ ± s)	48.2 ± 12.4		47.3 ± 9.6		0.745^a^
Gender (male/female)	23/10		15/11		>0.99^b^
Handedness	33 right-handed		26 right-handed		>0.99^a^
Tinnitus duration (months)	≥6 and ≤48		NA		NA
Tinnitus pitch	250 ∼ 8,000 Hz				NA
THI score	52.5 ± 44.3	37.3 ± 20.9	NA	NA	0.011^c^
ΔTHI score	15.3 ± 32.8	NA	NA	NA	NA
Laterality	2 right, 5 left, 26 bilateral				

*Data are presented as mean ± standard deviation for all variables except gender.*

*THI: Tinnitus Handicap Inventory.*

*ΔTHI = THIpre–THIpost.*

*NA: not applicable.*

*^a^Two-sample *t-*tests.*

*^b^Chi-square test.*

*^c^Paired-samples *t*-tests.*

### Sound Therapy and Clinical Evaluation

A TinniTest^®^ (TTS, 1000A, China) comprehensive tinnitus diagnosis and treatment instrument was used for psychoacoustic testing. SpeechEasy eMasker^®^ (Micro-DSP Technology Co., Ltd., China) was used to perform narrow-band sound therapy. We advised patients to use it in a quiet environment to achieve the best therapeutic effect. All of the enrolled tinnitus patients were examined for tinnitus loudness matching (L = loudness of tinnitus), pitch matching (Tf = tinnitus frequency), minimum masking level, and residual inhibition to characterize the patients’ tinnitus and prepare them for treatment. Narrow-band sound therapy was administered to the participants in the tinnitus group for 24 weeks, three times a day for 20 min each time. For each tinnitus patient, the loudness of sound for treatment was set as L-5 dB. The bandwidth was changed according to the center frequency, and the bandwidth was 1/3 octave (for example, Tf = 3 kHz, low cut = 3 kHz × 2-6/1, high cut = 3 kHz × 2^6/1^).

We also asked the patients to complete the Tinnitus Handicap Inventory (THI) to assess the severity of tinnitus before and after treatment. The primary outcome of this prospective study was the change in THI score after treatment. A reduction of at least 20 points in the THI score was considered effective treatment (Newman et al., 1998). No kind of sound was administered to the HC group during the study.

### Data Acquisition

The functional imaging data were obtained from the tinnitus patients at baseline (without any treatment) and at the end of therapy (24th week). The HC group was also scanned at baseline and at the 24th week. Images were obtained using a 3.0T MRI system (Prisma, Siemens, Erlangen, Germany) with a 64-channel phase-array head coil. During the scanning process, we used suitable foam padding to minimize head motion, and we used earplugs to reduce scanner noise. All the participants were asked to stay awake, close their eyes, breathe evenly, and try to avoid any specific thoughts. We used a conventional brain axial T1 sequence before the scans to exclude individuals with any visible brain abnormalities. We obtained resting-state functional images of all participants using an echo-planar imaging (EPI) sequence. We required the subjects to remain still during the scan time and not to meditate. The scanning parameters were as follows: 33 axial slices with a slice thickness = 3.5 mm and interslice gap = 1 mm, repetition time (TR) = 2000 ms, echo time (TE) = 30 ms, flip angle (FA) = 90°, bandwidth = 2368 Hz/Px, field of view (FOV) = 224 mm × 224 mm, and matrix = 64 × 64. The latter parameters resulted in an isotropic voxel size of 3.5 mm × 3.5 mm × 3.5 mm. The total number of volumes was acquired in 8.06 min.

### Pre-processing of Functional Data

Data pre-processing was performed using DPABISurf^1^, a surface-based RS-fMRI data analysis toolbox evolved from DPABISurf. DPABISurf uses the fMRIprep pipeline (Esteban et al., 2019) to pre-process the structural and functional MRI data and provides a set of statistical and viewing tools. The data pre-processing pipeline in the present study contained the following steps: (1) the initial five time points were discarded to allow for signal equilibration, (2) the data were converted into BIDS format (Gorgolewski and Poldrack, 2016), and then the fMRIPrep 1.5.0 docker was called. (3) Anatomical data pre-processing performed as follows: the T1-weighted (T1w) image was corrected for intensity non-uniformity (INU) with N4BiasFieldCorrection (Tustison et al., 2010) and was used as the T1w reference throughout the workflow. The T1w-reference was then skull-stripped with a Nipype implementation of the antsBrainExtraction workflow [from advanced normalization tools (ANTs)] (Avants et al., 2008) using OASIS30ANTs as the target template. Brain tissue segmentation of cerebrospinal fluid (CSF), WM, and GM was performed on brain-extracted T1w using fast (Zhang et al., 2001). Brain surfaces were reconstructed using recon-all (Dale et al., 1999). (4) Functional data pre-processing as follows: For each of the runs (resting-state) per subject, to achieve functional data pre-processing, the following steps were performed. First, a reference volume and its skull-stripped version were generated using a custom fMRIPrep methodology. The BOLD reference was then coregistered to the T1w reference using bbregister (FreeSurfer), which implements boundary-based registration (Greve and Fischl, 2009). BOLD runs were slice-time corrected using 3dTshift (Cox and Hyde, 1997). The BOLD time series were resampled to surfaces on the fsaverage5 space. (5) Nuisance regression was conducted as follows: the Friston 24-parameter model (Friston et al., 1996) was used to regress out head motion confounds. Additionally, mean framework displacement (FD) was used to address the residual effects of motion in group analyses (Jenkinson et al., 2002). Other sources of spurious variance (WM and CSF signals) were also removed from the data through linear regression to reduce respiratory and cardiac effects. Additionally, linear trends were included as a regressor to account for drifts in the BOLD signal. (6) Finally, data were filtered and smoothed as follows: a bandpass temporal filter (0.01–0.1 Hz) and spatial smoothing [full-width at half-maximum (FWHM) of 6 mm] were applied to the normalized functional images. After the above steps, we obtained cortical ALFF values.

### Statistical Analysis

Demographic data were compared through two-sample *t* tests and paired two-sample *t* tests using SPSS 19.0 software (SPSS, Inc., Chicago, IL, United States). *P* values < 0.05 were considered statistically significant. Longitudinal changes in THI scores were also analyzed by using paired two-sample *t* tests.

DPABISurf was used to pre-process the neuroimaging statistics. For cortical ALFF data, to determine the group × time interaction effect between the two groups and the two scans, the main effects of group (the tinnitus patient group and the HC group) and time (baseline and 24-week follow-up period), two-way mixed model analysis of variance (ANOVA) and *post hoc* analyses were performed. The brain regions showing significant time differences in the HC group were excluded (Wang et al., 2015). Cortical ALFF analyses were conducted using whole-brain analyses. A *P* value of less than 0.05 (*P* < 0.025 for each hemisphere) for cortical ALFF was considered statistically significant (Monte Carlo simulation corrected). We looked up tables of *P* values based on simulations in which a *Z* field was synthesized on the atlas surface. The tables were distributed in DPABISurf. The *P* value for the cluster was determined by indexing the table based on the size of the cluster, the threshold used to form the cluster, and an estimate of the global FWHM. Clusters were extracted separately for both hemispheres. In *post hoc* analyses, Student–Newman–Keuls (SNK) tests were used for pairwise comparisons. Pearson’s correlation analyses were further conducted to investigate the relationships between the change in cortical ALFF and 24 weeks of sound therapy of tinnitus patients [THI score (ΔTHI score = THIpre–THIpost)]. *P* < 0.05 was set as the threshold to determine significance. The cortical ALFF results were visualized with DPABISurf. Cortical ALFF was quantified from the T1w images using DPABISurf_V1.5 software. Pearson’s correlation analysis between the THI scores was performed using SPSS 19 software.

## Results

### Demographics and Behavioral Outcomes of Study Participants

Please see Table 1, and we used DPABISurf to analyze cortical ALFF in the brains of patients in this group before and after sound therapy. After head movement correction, no subjects were excluded (FD < 0.3). THI scores were acquired before and after sound therapy. Significant longitudinal decreases in THI scores were observed. The results are summarized in Table 1.

### Statistical Analysis Results

#### Group Differences in Amplitude of Low-Frequency Fluctuation

**FIGURE 1:**
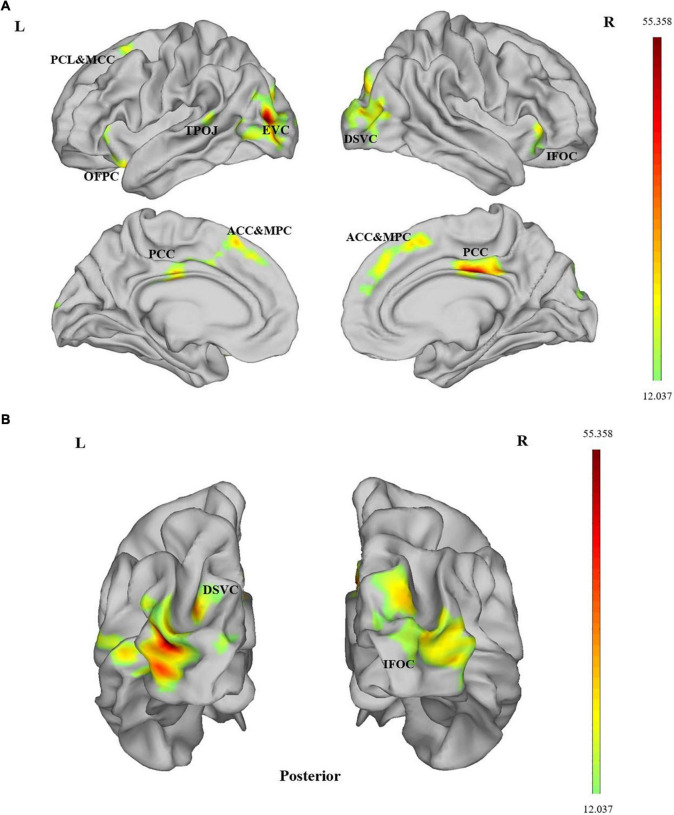
**(A,B)** ALFF differences were assessed by ANOVA. Compared among the four data sets, significant group × time interaction effects between the two groups (tinnitus patients and healthy controls) and two scans (at baseline and at the 24th week) on ALFF were observed at the bilateral DSVC, bilateral PCC, bilateral ACC and MPC, left TPOJ, left OPFC, left PCL and MCC, right IFOC and left EVC [*P* < 0.05 (*P* < 0.025 for each hemisphere) corrected by Monte Carlo simulation; L, left; R, right]. DSVC, dorsal stream visual cortex; PCC, posterior cingulate cortex; ACC and MPC, anterior cingulate and medial prefrontal cortex; TPOJ, temporo-parieto-occipital junction; OPFC, orbital and polar frontal cortex; PCL and MCC, paracentral lobular and mid cingulate cortex; IFOC, insular and frontal opercular cortex; EVC, early visual cortex.

As shown in Figure 1A and Table 2, there were differences in ALFF among the tinnitus patients before sound therapy (baseline), tinnitus patients after sound therapy (24 weeks), HC individuals at baseline and HC individuals after 24 weeks. The relevant brain regions included the bilateral dorsal stream visual cortex (DSVC), bilateral posterior cingulate cortex (PCC), bilateral anterior cingulate and medial prefrontal cortex (ACC and MPC), left temporo-parieto-occipital junction (TPOJ), left orbital and polar frontal cortex (OPFC), left paracentral lobular and mid cingulate cortex (PCL and MCC), right insular and frontal opercular cortex (IFOC), and left early visual cortex (EVC) [*P* < 0.05 (*P* < 0.025 for each hemisphere) corrected by Monte Carlo simulation] (Figures 1A,B).

**TABLE 2:** Difference in cortical ALFF of the left and right hemisphere between the two groups (tinnitus patients, healthy controls) and two scans (at baseline, at the 24th week).

**Brain regions**		**HCP**	**Cluster Size (mm^2^)**	**Coordinates MNI**			**Peak *F* score**
				***x***	***y***	***z***	
Dorsal stream visual cortex	L	16	336	−24	−81	23	34.64
	R	13	2252	31	−89	11	25.80
Posterior cingulate cortex	L	14	241	−6	−17	32	32.15
	R	32	668	6	−19	32	48.79
Anterior cingulate and medial prefrontal cortex	L	63	314	−7	23	53	29.17
	R	63	708	8	20	50	24.41
Temporo-parieto-occipital junction	L	139	237	−63	−42	11	20.51
Orbital and polar frontal cortex	L	94	385	−26	14	−20	25.88
Paracentral lobular and mid cingulate cortex	L	41	175	−11	6.1	37	18.51
Insular and frontal opercular cortex	R	111	500	29	27	0	21.78
Early visual cortex	L	6	2265	−33	−82	8	55.36

*Statistically differences in cortical ALFF were defined as *P* < 0.05 (*P* < 0.025 for each hemisphere), Monte Carlo Simulation corrected after correcting for age, sex, education, and the head motion.*

*MNI, Montreal Neurological Institute; HCP, Human Connectome Project.*

### *Post hoc* Analyses

**FIGURE 2:**
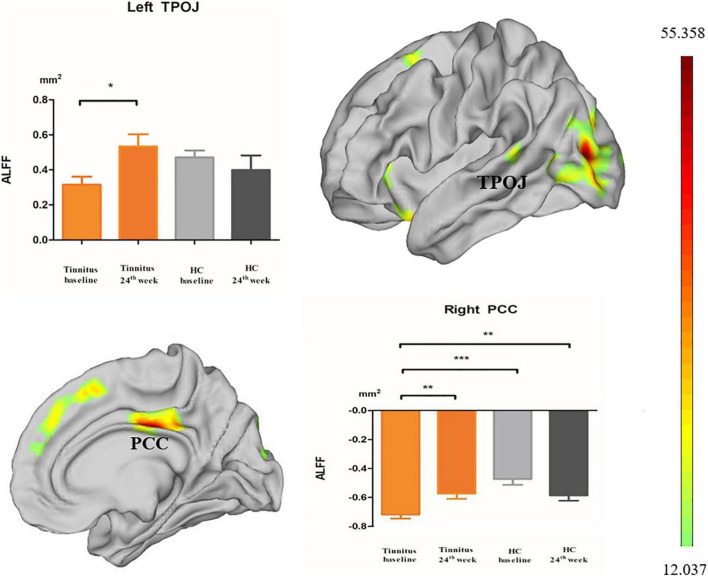
*Post hoc* analyses showed differences in ALFF among the baseline, 24-week sound treatment, HC baseline and HC 24-week groups in both hemispheres. *P < 0.05, **P < 0.01, ***P < 0.001.

Because other brain regions showing time differences in the HC group were excluded, only the left TPOJ and right PCC were retained for further analysis. *Post hoc* analysis in this study confirmed that patients with tinnitus all had decreased ALFF in related brain regions. ALFF was significantly decreased in the left TPOJ and right PCC of participants in the tinnitus baseline group compared to participants in the HC baseline group and HC 24-week group; also, there were no differences between the two HC groups (Figure 2). The 24-week sound therapy tinnitus group demonstrated significantly higher ALFF in the left TPOJ and right PCC than in the tinnitus baseline group (Figure 2).

Compared with the HC baseline group and the HC 24-week group, the 24-week sound therapy tinnitus group demonstrated slightly lower or higher ALFF in the left TPOJ and right PCC, and there were no differences between the 24-week sound therapy tinnitus group and either of the HC groups.

In *post hoc* analysis, there were no differences between the tinnitus baseline group and the 24-week sound therapy tinnitus group in the left DSVC, left OPFC, right ACC and MPC, right IFOC, and left EVC.

### Correlation

Decreased THI scores and ALFF changes in the abovementioned brain regions were not correlated.

## Discussion

This is the first study to demonstrate cortical functional abnormality involving ALFF in patients with tinnitus. In this study, we analyzed the changes in cortical ALFF of tinnitus patients before and after 24 weeks of sound therapy. Cortical ALFF changes in the brain were found in patients from before and after longer-term sound therapy after multiple comparison corrections were conducted. Changes were mainly in the bilateral DSVC, bilateral PCC, bilateral ACC and MPC, left TPOJ, left OPFC, left PCL and MCC, right IFOC and EVC. Importantly, cortical ALFF changes in the left TPOJ and right PCC remained significant after *post hoc* analysis. These brain regions are mainly concentrated in the auditory cortex, visual cortex and PCC, which are closely related to the abnormal brain local neural activity associated with tinnitus. Our data confirmed our hypothesis that sound therapy could gradually restore brain function, especially local neural activity, to a relatively normal range.

We included patients whose tinnitus duration less than 48 months. Because of shorter duration may help with treatment effects (Schoisswohl et al., 2021). In our study, we observed that the 24-week sound therapy tinnitus group demonstrated significantly higher ALFF in the right PCC than in the tinnitus baseline group. As an important nucleus in the limbic system, the cingulate gyrus is widely connected with other areas of the central nervous system; moreover, it participates in various functions, such as regulating emotions, learning, and cognition (Fan et al., 2020). The PCC is an important structure of the default mode network (DMN) (Raichle and Snyder, 2007; Lan et al., 2021) and belongs to the frontal-parietal-limbic network, which has been regarded as a specific distress network in tinnitus (Husain, 2016). Abnormalities in the PCC are usually related to cognitive impairment, including memory function, attention, and problems in maintaining a balance between internal and external thinking (Leech and Sharp, 2014). The complete symptomology of tinnitus includes at least three factors: sensation, emotion, and cognition. Research on tinnitus has shown that abnormal changes in the cingulate cortex are involved in the process of tinnitus and play a key role in noise canceling, cognitive experiences and emotional experiences in tinnitus patients (Fan et al., 2020). “Dysfunctional noise canceling mechanism” has been conceptualized (De Ridder et al., 2012). A previous study found that some noise-canceling related effects in the subcallosal area (Rauschecker et al., 2010). The results of another study may designate the role of the rostral ACC as the core of the descending noise cancelation system (Song et al., 2015). These results all suggested the possibility of tinnitus perception modulation by neuromodulatory approaches to change the activity of above brain regions.

According to a previous report, the PCC undergoes significant changes in functional activity that ameliorate patient’s pain after tinnitus treatment (Krick et al., 2017). In this study, the PCC was considered a hub brain region of the DMN, and ALFF became normal after sound therapy, reflecting that activation of the PCC was enhanced after treatment. This result was consistent with previous research on volume-based ALFF before and after treatment (Zhang A. et al., 2019). The results also showed that there were no differences between the 24-week sound therapy tinnitus group and HC groups in the PCC, showing that after sound therapy, cognition, attention, emotion and other related functions may be restored to a certain extent.

The HCP template identified the TPOJ as a strip of cortex bounded by the auditory, lateral temporal, inferior parietal and occipital cortices (Glasser et al., 2016). Although the TPOJ is not well known by many people, it contains the auditory cortex (lateral temporal) and visual cortex (occipital cortex), forming a bridge between advanced auditory and advanced visual areas (Glasser et al., 2016), which is closely related to tinnitus. Some previous volume-based RS-fMRI studies have shown that the functional connectivity between the visual network and the auditory network of tinnitus patients is negatively correlated. Tinnitus may also cause a decrease in spontaneous neural activity in the visual area (Burton et al., 2012; Maudoux et al., 2012). Through surface-based RS-fMRI, our research also inferred that the function of the auditory and visual cortices of patients with tinnitus maybe restored after long-term treatment.

In this study, we used surface-based ALFF analysis instead of volume-based ALFF analysis. Compared with surface-based methods, volume-based processing steps, especially smoothing and registration, significantly degrade cortical area localization; moreover, the spatial localization effect of the latter is only 35% of the best spatial positioning of the former (Coalson et al., 2018). As another neural activity indicator, cortical ReHo was demonstrated to be more specific to the intrinsic functional organization of the cortical mantle and had higher test–retest reliability (Zhang B. et al., 2019). However, in the tinnitus field, there is no research on the use of ALFF in the cortex, especially for comparisons before and after treatment. Therefore, our results proved the effectiveness of cortical functional imaging after tinnitus therapy to a certain extent and could more accurately reflect local functional changes at the cortical level. In addition, we used 24 weeks of sound therapy to evaluate patients after a relatively long period of time in this study. Compared with the shorter treatment period of 12 weeks used in previous studies (Wei et al., 2020; Lv et al., 2021), 24 weeks of treatment was more effective. With the extended treatment time, we obtained more detailed cortical information.

### Limitations

First, there was no relationship between decreased THI scores and ALFF changes in tinnitus patients, and only left TPOJ and right PCC showed differences between the tinnitus baseline group and the 24-week sound therapy tinnitus group in the *post hoc* analysis; this outcome may have been due to the small sample size. In future studies, we need to further expand the sample size to verify the correlation between the differences observed. Second, few studies have reported treatment effects for tinnitus at the surface-based level, especially with ALFF. There are few related papers for our reference. Therefore, we need to combine other surface-based indicators, such as ReHo, degree centrality, and functional ALFF (fALFF), to study their significance more comprehensively. Third, the treatment time of 24 weeks should be gradually increased while considering the patient’s tolerance. After all, achieving a longer period of clinical treatment is conducive to the recovery from tinnitus.

## Conclusion

Longer-term sound therapy changed cortical ALFF in left TPOJ and right PCC. Surface-based RS-fMRI can provide more subtle local functional activity to explain the mechanism of tinnitus treatment, and these brain regions could serve as potential targets in the brain for neuroimaging evaluation of sound therapy in tinnitus patients. Notably, long-term sound therapy had a normalizing effect on tinnitus patients. Future follow-up studies in a larger cohort may elucidate further meaning of the changes in ALFF value after tinnitus.

## Data Availability Statement

The original contributions presented in the study are included in the article/supplementary material, further inquiries can be directed to the corresponding author/s.

## Ethics Statement

The studies involving human participants were reviewed and approved by this research involved human participants. All authors have declared that this research was approved by the Institutional Review Board (IRB) of Beijing Friendship Hospital, Capital Medical University, Beijing, China. The patients/participants provided their written informed consent to participate in this study. Written informed consent was obtained from the individual(s) for the publication of any potentially identifiable images or data included in this article.

## Author Contributions

XW designed the experiments, performed the statistical analysis, and wrote the manuscript. QC conducted the statistical analysis. PZ contributed to the manuscript revision. HL, ZW, and CL collected the data. SG, ZY, and ZcW were guarantors of this work. HL and ZcW have full access to all the data in the study and take responsibility for the integrity of the data and the accuracy of the data analysis. All authors contributed to the article and approved the submitted version.

## Conflict of Interest

The authors declare that the research was conducted in the absence of any commercial or financial relationships that could be construed as a potential conflict of interest.

## Publisher’s Note

All claims expressed in this article are solely those of the authors and do not necessarily represent those of their affiliated organizations, or those of the publisher, the editors and the reviewers. Any product that may be evaluated in this article, or claim that may be made by its manufacturer, is not guaranteed or endorsed by the publisher.

## Footnotes

1
http://rfmri.org/DPABISurf
